# Safety assessment of all-steel-type attached lifting scaffold based on grey Euclidean theory

**DOI:** 10.1371/journal.pone.0238074

**Published:** 2020-08-27

**Authors:** Yijun Liu, Qin Li, Wenlong Li, Huimin Li, Xingwang Pei

**Affiliations:** 1 School of Civil Engineering, Xi’an University of Architecture and Technology, Xi’an, Shaanxi, China; 2 School of Architecture and Urban Planning, Beijing University of Civil Engineering and Architecture, Beijing, China; Wuhan University, CHINA

## Abstract

To reduce the incidence of safety accidents during the construction of all-steel-type attached lifting scaffolds and address the research gaps in related fields, in this study a theoretical model of trajectory crossing accidents was coupled with an analysis of similar safety accidents to determine the causes of accidents in the construction of high-rise buildings using steel-type attached lifting scaffolds. To do so, a safety evaluation index system covering all steel-type attached lifting scaffolds that comprises five first-level indicators and 17 second-level indicators was established. The first-level indicators cover three risk dimensions: unsafe human behavior (personal operations), unsafe conditions (material performance, structural calculation, components and connections), and lack of management (safety management). A combined multi-agent-based modeling (MABM) method and structural entropy weight were used to calculate a comprehensive weight for better alignment of the weight calculation results with objective laws. A safety assessment model for an all-steel-type attached lifting scaffolding was constructed using grey relative Euclidean weighted correlation theory to enable the calculation of a grey-to-Euclidean weighted correlation degree that directly correlates with the degree of security. Using the established assessment model, four projects were subjected to a safety evaluation, with the results validating the model by revealing that its output was consistent with the actual security situation.

## 1. Introduction

All-steel-type attached lifting scaffoldings are one of the “Top Ten New Construction Technologies” [1] promoted by the Ministry of Housing and Urban-Rural Development of the People’s Republic of China in recent years. These scaffoldings are superior to traditional scaffolding in terms of durability, reduced energy use and environmental protection, and rapid turnover, and they have been widely used in engineering projects in recent years. However, the use of new types of equipment has resulted in a technological revolution and new requirements for safety management workers. On September 10, 2011, a high-rise attachment lifting scaffolding accident at a construction site in Xi’an killed ten and seriously injured two people. In August 2016, a typhoon caused the partial falling of an all-steel-type attached lifting scaffold at a construction site in Shenzhen with, fortunately, no related injuries. On March 21, 2019, an all-steel-type attached lifting scaffold dropped at a construction site in Yangzhou, killing six and injuring 11 people. These incidents illustrate the danger of safety accidents caused by all-steel-type attached lifting scaffolds and the need for effective control measures to ensure the safe deployment of all-steel climbing scaffolds by China’s construction industry [2].

To date, research on the safety management of attached lifting scaffolds in China has resulted in the successive promulgation of standards such as JGJ 202–2010, “Safety Technical Specifications for Construction Tool Scaffolding” [3] and GB 51210–2016, “Uniform Standards for Safety Technology for Construction Scaffolding” [4]. Recently, the Ministry of Housing and Urban-Rural Development of the People’s Republic of China (MOHURD) issued a number of documents and interim regulations, including the “Emergency Notice on Strengthening the Management of the Use of Integrated Lifting Scaffolding in Building Construction” [5]. These frameworks have effectively promoted the safety management of all-steel-type attachment lifting scaffolds.

From a research perspective, W.K. Yu et al. [6] conducted a systematic study on the structural performance of multi-layer portal-type combined steel supports based on experiments and numerical studies, while Jui-Lin Peng et al. [7] studied the effects of eccentric loads on the steel scaffolding system at a construction site. Zhang et al. [8] conducted the first analysis of the effects of structural strength and safety influence factors on all-steel-type scaffolding to identify the corresponding safety control points. Xu Yan [9] applied the accident tree method to analyze scaffold frame structure, design, quality of fasteners and steel pipes, unsafe human behavior, management defects, etc., and to explore effective control measures for these. Using a semi-rigid steel support structure design as an example, Hao Zhang and Kim J.R. Rasmussen [10] compared three different advanced design analysis methods and discussed the use of advanced analysis in developing a system-based design method. Hu Hong et al. [11] used an example project to analyze the primary causes of accidents in terms of the logical relationships between basic events and performed a qualitative analysis to determine the key approaches to accident prevention and provide a basis for preventing scaffolding engineering overturn and collapse accidents. Chengwu Wang et al. [12] constructed a scaffolding security management system model and described its system functions and framework using Unified Modeling Language use case diagrams, class diagrams, and deployment diagrams. Hu Hailong [13] elaborated safety management and control measures as a series of processes including source control, process monitoring, and aerial disassembly of an attached lifting scaffold. Liu Qingqing [14]analyzed the safety of overhanging scaffolding with early warning management systems. Chen Shu et al. [15] systematically analyzed the accident induction mechanism of scaffolding operations and further detailed its influencing factors and the logical relationship among them to establish a scaffolding accident tree. Wang Huihai [16] analyzed the basic structure of all-steel-type attached lifting scaffolding and proposed a series of safety management measures. Guo Weichao et al. [17] used a project in Hefei China Resources Vientiane City as an example to study the climbing technology of all-steel-type attached lifting scaffolds at the shrinkage of the structure under complex conditions. Wang Sunmeng et al. [18] constructed a safety evaluation model of cantilever scaffolding to comprehensively evaluate its safety level at a construction site. Xin Bai [19] used a project in Handan as an example for designing and validating a new type of attached scaffold lifting device via simulation using finite element static analysis. Zhu Zhengquan [20] analyzed the safety-influencing factors of an attached scaffold and the dynamic response of the scaffold during the climbing process and carried out numerical simulation using finite element software to develop a method for strengthening the overall stability of the frame. Hongbo Liu et al. [21] conducted a systematic experimental and analytical study of the stability behavior of the horizontal pipe in the upper part of a steel pipe fastener bracket under construction load. Mou Yangyang [22] introduced the basic structure of an all-steel-type attached lifting scaffolding and proposed a number of safety management measures. Hu Shijun et al. [23] used ANSYS software to establish a finite element model of an attached lifting scaffold, analyzed its structural modals under different working conditions, and combed its natural frequencies and mode shapes to produce a foundation for succeeding safety analyses. Jia Li et al. [24] analyzed the instability mechanisms and failure modes of ultra-high fastener steel pipe full-scaffolding scaffolding under uniform load by applying static tests to seven different models and also investigated pole step and fastener tightening torque. Gian Paolo Cimellaro et al. [25] carried out finite element simulations of three types of steel structure supports under different loading conditions and proposed empirical formulas to identify the critical loads of different types of supports. Tian Baoji et al. [26] developed an attached lifting scaffold that combines left-right and pitch obliques and has significant potential to improve high-rise building construction safety. Yin Xinxin [27] applied rough set theory to develop the first safety evaluation model for a conventional attached scaffold as a theoretical reference for the safety management of attached scaffolds. Yang Zheming [28] discussed the construction, deployment, installation, and lifting measures of an all-steel-type attached lifting scaffold based on an example of a super high-rise office building. Tan Ming [29] conducted a thorough analysis of scaffolding and wall fittings, discussed the state-of-the-art of and future research and development directions for wall fittings, and clarified the safety technology and management concepts relevant to such fittings. Wang Changsheng et al. [30] used a residential district project in Chengdu as an example to propose corresponding safety construction measures for an attached pull-up cantilever scaffold. Du Dongli [31] explored and analyzed the technical application and safety management of attached lifting scaffoldings in building construction. Hu Rongchuan [32] discussed building scaffolding construction safety construction management countermeasures from multiple aspects. Zi Xuebin et al. [33] analyzed the safety management of cantilever scaffolding in construction. Using the characteristics of all-steel-type attached lifting scaffolding as a basis for discussion, Yi Xi [34] examined construction techniques such as equipment installation, removal from air, and safety management. Ma Xing [35] analyzed the technological principles and characteristics of all-steel-type attached lifting scaffolding and explored its primary structural and applicational advantages. Xu Jianlong [36] proposed a new type of all-steel integrated scaffold with a changeable tilt angle that could climb the complex hyperbolic gradient octagonal facade of a specific building project. Wu Chengchen [37] assessed the four major systems that affect the safety of attached lifting scaffolding and used the results to propose safety improvement measures.

Overall, the literature on the safety management of conventional scaffolding construction is relatively mature. Past research on all-steel-type attached lifting scaffolds has primarily focused on equipment research and development, process improvement, stability analysis, structural system design, etc. However, there have been few studies on the safety evaluation of all-steel-type attachment lifting scaffolds from either a theoretical or practical perspective. In view of this, and considering the great relevance of factors that influence safety and the ambiguity and incompleteness of the available information, this paper attempts to assess the safety of all-steel-type attached lifting scaffolding through an evaluation model based on grey relative Euclidean weighted relevance theory. The results are expected to provide a theoretical basis for the safety management of all-steel-type attached lifting scaffolds in the Chinese construction industry and a reference for related safety management practitioners.

## 2. Methods

### 2.1. Research area

In October 2011, the Office of Work Safety of the State Council was notified of a "9.10" major accident involving a falling attachment scaffold at the Kaixuan Building construction site in Xi'an, Shaanxi Province, China. According to an analysis by relevant departments, the accident was caused by the failure of the construction unit to evacuate workers from the rack while implementing the overall lifting of the attached scaffolding. In this case, the load-bearing members were dismantled in violation of the rules, causing the rack to fall out of balance. In a recent major accident on May 23, 2020, a formwork support collapse occurred at an illegal land construction project in Mabugang Town, Longchuan County, killing eight people and injuring one person. These accidents reveal that there remain weak links in the safety supervision of engineering construction projects. In particular, there are loopholes in the implementation of responsibilities, technical support, and measures at construction sites involving shortfalls in terms of command coordination, construction organization, job delivery, and safety protection.

**Fig 1:**
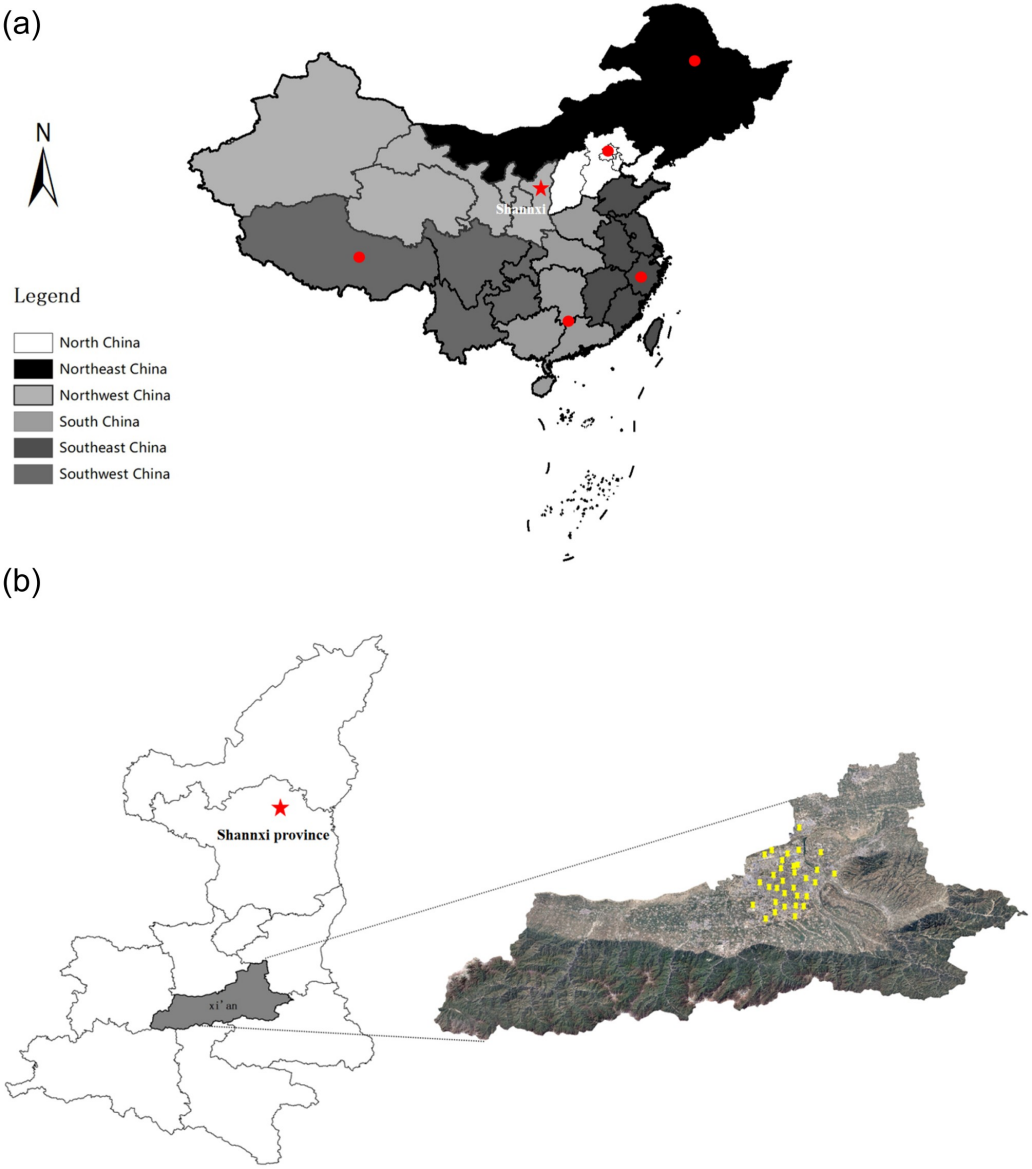
Location of research. (a) survey area map; (b) distribution of survey sites in Xi’an, Shaanxi province.

Our research team visited many provinces and cities to investigate project sites at which all-steel-type lifting scaffolds are used. Discussions were carried out with relevant professional and technical personnel to obtain an in-depth understanding of the use, maintenance, and performance of this type of scaffolding in construction. The area surveyed over the course of this study is shown in Fig 1a. In Fig 1b, Xi'an is taken as an example to show the distribution of surveyed locations.

### 2.2. Selection of indicators

#### 2.2.1 Primary selection of indicators

All-steel-type attached lifting scaffoldings have been increasingly deployed in recent years to meet the needs of high-rise building structure construction and the requirements of energy saving and emissions reduction. Such scaffoldings are widely used in construction projects in major cities across the country. Unlike conventional attached lifting scaffolding fabricated using circular steel pipes, all-steel-type attached lifting scaffolds require sizing, tooling, and mechanization and have distinctive characteristics in terms of material selection, component connection, structural calculation, maintenance, etc., that differ from those of conventional scaffolding.

**Fig 2:**
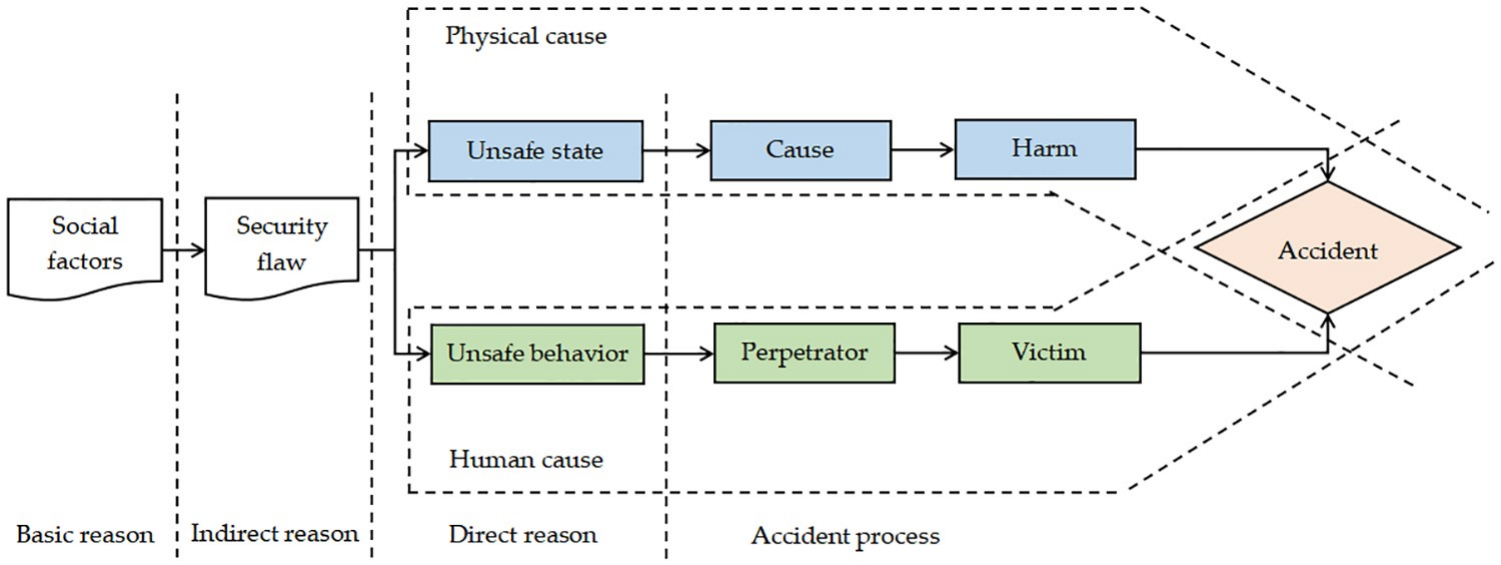
Theoretical model of trajectory-crossing accident.

By applying the cause theory of trajectory crossing accidents [2], in this study the influencing factors that can compromise the operational safety of all-steel-type attached lifting scaffolds were comprehensively analyzed and accurately identified to clearly determine the essential causes of safety accidents. Trajectory intersection theory is used to study the direct and indirect causes of accidents. Security accidents occur as the result of the sequential development of many interrelated events, which can be categorized into two primary categories: people- and condition- (including the environment) related. When unsafe behaviors and conditions come into contact, safety accidents will occur. Unsafe human behavior can be attributed to physical, psychological, environmental, behavioral, and other factors. An unsafe state can occur at any stage along a trajectory of physical factors applying to a production process; a theoretical model of trajectory crossing accident causes is shown in Fig 2.

An analysis of the causes of past safety accidents involving all-steel-type and similar conventional attached lifting scaffolds reveals the following: accident occurrence is related not only to the unsafe behavior of people (illegal operations, overly hasty dismantling, etc.) but also to unsafe machinery and equipment states (defects in materials, poor structural stability, etc.) and a lack of management linkage (unsound systems, insufficient maintenance, etc.). Accident occurrence is also closely related to the interaction among various risk factors.

In addition to the theoretical modeling of the causes of trajectory-crossing accidents and the basic principles of index system construction, on-site investigation and literature analysis can be used to identify risk factors along three dimensions: unsafe human behavior (personnel operation), unsafe material state (material performance, structural calculation, components and connections), and lack of management (safety management). In this study, five first-level indicators were summarized and 28 safety influencing factors were initially determined as follows:

- Material properties:
  - square tube wall thickness (B_1_);
  - square tube section size (B_2_);
  - selection of electric hoist (B_3_);
  - initial defect (B_4_).
- Structural calculation:
  - square tube strength (B_5_);
  - square tube deformation (B_6_);
  - square tube stability (B_7_).
- Components and connections:
  - location of wall pieces (B_8_);
  - vertical distance, horizontal distance, and height of the frame (B_9_);
  - scaffolding position and constraints (B_10_);
  - arrangement of inclined rod and platform plate (B_11_);
  - setting of dense mesh (B_12_);
  - void density of dense mesh (B_13_);
  - reliability of anti-falling device (B_14_).
- Safety management:
  - safety management regulations (B_15_);
  - safety education and training work (B_16_);
  - safety cost input (B_17_);
  - site management and safety inspection (B_18_);
  - division of safety responsibility system (B_19_);
  - equipment maintenance (B_20_);
  - equipment emergency plan system (B_21_).
- Personnel operation:
  - installation of equipment (B_22_);
  - use of equipment (B_23_);
  - removal of equipment (B_24_);
  - skill level (B_25_);
  - safety protection (B_26_);
  - technical disclosure (B_27_);
  - operating platform erection (B_28_).

#### 2.2.2 Screening of indicators

To ensure that the indicators were targeted and representative, some of the initial indicators were eliminated through expert consultation. This was done by organizing all 28 indicators into a table and eliminating or merging several based on the advice of relevant technical personnel in construction and scaffold contracting units. The removed indicators and reasons for removal are listed in the Table 1.

**Table 1:** Eliminated indicators and reasons for removal.

Index	Reason for rejection
B_13_	The design of the dense mesh should meet the requirements of the national standard JGJ 130–2011 “Safety Technical Specification for Fastener-type Steel Pipe Scaffolding for Construction” [38]. As this is not used to evaluate the safety of scaffoldings, it was rejected.
B_17_	As special economic analysis of cost inputs, this indicator was eliminated.
B_19_	Recommended for merging with “safety management regulations” and changed to more representative “establishment of safety management system”.
B_21_	This system should be used to conduct specific scaffolding analysis following the safety assessment to formulate systematic emergency measures. The focus should be on avoiding safety accidents and the losses caused by accidents.
B_25_	Combined with “safety education and training work” to make the assessment more targeted.
B_26_	
B_27_	Combined with “site management and safety inspection” to make the assessment is more targeted.
B_28_	This should be considered as part of the preparatory work for construction safety assessment.

#### 2.2.3 Determination of indicators

Questionnaires were developed to further examine the relationship between the 20 indicators that survived the screening and scaffold construction. Two variable settings—quantitative and qualitative—were used in the survey questionnaire. The data sources for the quantitative questions included documents related to the use of scaffolding in project construction. The qualitative questions were designed to capture the subjective feelings of the respondents and were scored using the Likert 5 scoring method, in which scores of 1 to 5 (“very unimportant” to “very important,” respectively) are assigned.

Out of a total of 268 questionnaires distributed, 201 were recovered. Following screening, 169 valid questionnaires remained, corresponding to a questionnaire validity rate of 84.0%. SPSS26.0 software was used to analyze the responses.

1. Reliability analysis
  Reliability represents the consistency and stability of a scale, and reliability coefficients can also be used as indicators of tested homogeneity in the analysis of scale items based on the application of Cronbach’s α coefficient. In this study, “Cronbach's α coefficient after item deletion,” or the overall reliability coefficient of the overall scale following item deletion, was used as a reliability indicator. An indicator that is significantly higher than the original Cronbach's α coefficient suggests that the item is not homogenous with other items and can be considered for deletion according to the actual situation. The results of the indicator elimination process are summarized as follows:
  - First round: The overall Cronbach α coefficient of the scale was 0.897, indicating that the reliability of the scale was very high. The Cronbach α coefficient of B_2_ was 0.900, indicating that the measured attribute of this item was inconsistent with the existence of other items; therefore, it was eliminated.
  - Second round: After excluding B_2_, the overall Cronbach α coefficient of the scale was 0.896 and the Cronbach α coefficient of B_3_ was 0.897; therefore, it too was eliminated.
  - Third round: After excluding B_3_, the overall Cronbach α coefficient of the scale was 0.892, and the Cronbach α coefficient of B_10_ was 0.892. After consulting relevant experts, B_10_ was removed.
  After B_2_, B_3_, and B_10_ were eliminated, the overall Cronbach α coefficient of the scale were 0.892, which, as it was higher than 0.8, indicated that the reliability of the total scale was very high [39]. The Cronbach α coefficients of all indicators were above 0.8, indicating that the correlation between the indicators was high. The final results of the reliability analysis are listed in Table 2.
2. Validity analysis
  The Kaiser-Meyer-Olkin (KMO) test is used to assess the sampling accuracy of data. According to Kaiser (1974), the KMO should be between zero and one, with values closer to one being more suitable for factor analysis. The Bartlett sphere test is used to test whether variables are independent of each other; if the P-value of a test reaches a significance level of P < 0.05, the variable is suitable for factor analysis [40]. The KMO and Bartlett sphere tests were applied to the remaining 17 items, with the results listed in Table 3.
  The 17-item KMO (0.85) and the Bartlett’s sphericity P (0.000) were higher and lower than their respective significance levels of 0.7 and 0.05, indicating that the scale was suitable for factor analysis [41].
  Following the successful KMO and Bartlett sphere tests, principal component analysis was applied for factor analysis, with eigenvalues greater than one set as the extraction principle and the Kaiser standardized maximum variance method applied for orthogonal rotation. The post-rotation factor analysis results are listed in Table 4.
  It is seen from Table 4 that, following orthogonal rotation, a total of four effective factors with eigenvalues greater than one were obtained. The variance contribution rates of the four factors were 21.115, 14.616, 13.390, and 12.524%, respectively. The cumulative variance contribution rate was 61.644%, indicating that the four factors explained 61.644% of the information on the scale and reached the minimum standard of 60%. Moreover, the factor load of each item along its respective dimension was greater than 0.5, indicating that the extracted factors could be accepted [42].
  The results of the above factor analysis indicate that the scale used in this study had a high construction validity and, therefore, that the index system could be used to evaluate the construction safety of all-steel-type attached lifting scaffolds. The refined 17 influencing factors were then used to construct an all-steel-type attached lifting scaffold safety evaluation index system (Table 5). Interpretations of the respective indicators are listed in Table 6.

**Table 2:** The results of reliability test.

Indicator	Title	CITC	Cronbach α CADT	Cronbach α CTT
Material properties	B_1_	0.416	0.890	0.892
	B_2_	0.474	0.888	
Structural calculation	B_3_	0.392	0.890	
	B_4_	0.634	0.883	
	B_5_	0.522	0.886	
Components and connections	B_6_	0.504	0.887	
	B_7_	0.435	0.891	
	B_8_	0.543	0.886	
	B_9_	0.481	0.888	
	B_10_	0.622	0.883	
Safety management	B_11_	0.574	0.885	
	B_12_	0.671	0.881	
	B_13_	0.667	0.881	
	B_14_	0.700	0.879	
Personnel operation	B_15_	0.541	0.886	
	B_16_	0.548	0.885	
	B_17_	0.476	0.888	

CITC: Corrected items total correlation; Cronbach α CADT: Cronbach α coefficient after deleting terms; Cronbach α CTT: Cronbach α coefficient of total table.

**Table 3:** KMO and Bartlett inspection results.

Kaiser-Meyer-Olkin metric with sufficient sampling		.855
Bartlett sphericity test	Approximate chi-square	705.207
	Degrees of freedom	136
	Distinctiveness	.000

**Table 4:** Post-rotation factor analysis table.

	Ingredient			
	1	2	3	4
B_13_	.851			
B_10_	.721			
B_8_	.700			
B_14_	.636			
B_12_	.616			
B_2_	.572			
B_4_	.537			
B_6_		.734		
B_15_		.661		
B_5_		.536		
B_1_			.817	
B_3_			.688	
B_11_			.588	
B_9_			.543	
B_16_				.680
B_7_				.649
B_17_				.602
Eigenvalues	3.590	2.485	2.276	2.129
Variance contribution rate (%)	21.115	14.616	13.390	12.524
Cumulative variance contribution rate (%)	21.115	35.731	49.121	61.644

**Table 5:** Framework of safety evaluation.

Criterion	Indicator	Literature
Material properties A_1_	Wall thickness of square tube (A_11_)	W.K. Yu et al. (2004); Wang Sunmeng (2016); Liu Qingqing (2015); Chen Shu (2015); Hu Hong (2014)
	Initial defect (A_12_)	Jui-Lin Peng et al. (2009); Wang Sunmeng (2016); Hu Rongchuan (2019); Hu Hailong (2014)
Structural calculation A_2_	Square tube strength (A_21_)	Hao Zhang and Kim J.R. Rasmussen(2013); Liu Qingqing (2015); Jia Li (2017); Chen Shu (2015);
	Square tube deformation (A_22_)	Jui-Lin Peng et al.(2009); Liu Qingqing (2015); Ma Xing (2019); Wang Chengwu (2014)
	Square tube stability (A_23_)	Ma Xing (2019); Jia Li (2017); Hu Hong (2014); W.K. Yu et al.(2004)
Components and connections A_3_	Location of wall pieces (A_31_)	Gian Paolo Cimellaro et al.(2017); Wu Chengchen (2020); Guo Weichao (2016); Wang Sunmeng (2016); Tan Ming (2018)
	Vertical distance, horizontal distance, and height of the frame (A_32_)	Hao Zhang and Kim J.R. Rasmussen(2013); Wu Chengchen (2020); Wang Sunmeng (2016); Wang Sunmeng (2016); Liu Qingqing (2015)
	Arrangement of inclined rod and platform plate (A_33_)	Wu Chengchen (2020); Guo Weichao (2016); Wang Sunmeng (2016); Jia Li (2017)
	Settings of dense mesh (A_34_)	Hongbo Liu et al.(2016); Liu Qingqing (2015); Ma Xing (2019); Wang Chengwu (2014)
	Reliability of anti-falling device (A_35_)	Wu Chengchen (2020); Xu Jianlong (2019); Hu Hailong (2014); Hu Hong (2014)
Safety management A_4_	Establishment of safety management system (A_41_)	Du Dongli (2019); Guo Weichao (2016); Wang Sunmeng (2016); Liu Qingqing (2015); Hu Rongchuan (2019); Zi Xuebin (2019)
	Safety education and training work (A_42_)	Wang Changsheng (2019); Du Dongli (2019); Wang Sunmeng (2016); Hu Rongchuan (2019);
	Site management and safety inspection (A_43_)	Guo Weichao (2016); Wang Sunmeng (2016); Liu Qingqing (2015); Zi Xuebin (2019); Yi Xi (2019)
	Equipment maintenance (A_44_)	Guo Weichao (2016); Liu Qingqing (2015); Zi Xuebin (2019); Tan Ming (2018)
Personnel operation A_5_	Installation of equipment (A_51_)	Wang Changsheng (2019); Du Dongli (2019); Hu Rongchuan (2019); Yang Zheming (2019); Yi Xi (2019)
	Use of equipment (A_52_)	Wu Chengchen (2020); Guo Weichao (2016); Yang Zheming (2019)
	Removal of equipment (A_53_)	Wang Changsheng (2019); Du Dongli (2019); Yang Zheming (2019); Yi Xi (2019); Xu Jianlong (2019)

**Table 6:** Indicator interpretation.

Index	Interpretation
*A*_11_	The error in wall thickness shall be within the specified standard range.
*A*_12_	The frequency of steel tube reuse is reduced and the steel tube is newer and has no abnormalities.
*A*_21_	The strength calculation value is within the allowable range of the specification.
*A*_22_	The calculated value of deflection is within the allowable range of the specification.
*A*_23_	The stability calculation value is within the allowable range of the specification.
*A*_31_	According to the professional construction standard setting, the specification design standard is met.
*A*_32_	The error is within the acceptable range and the value is small.
*A*_33_	Quantity and location meet specification requirements.
*A*_34_	The location and specification meet the requirements of the construction plan.
*A*_35_	Location and quantity meet specification requirements.
*A*_41_	The safety system is perfect and the safety management organization is complete.
*A*_42_	Operational personnel have strict training in the use of the management system.
*A*_43_	Section acceptance, full-time responsible management person is on site.
*A*_44_	Regular maintenance and replacement of components.
*A*_51_	Procedures and methods are set up in accordance with the requirements of the construction scheme.
*A*_52_	According to the construction plan.
*A*_53_	Demolition sequence performed according to the construction requirements.

### 2.3 Methodology

#### 2.3.1 Grey Euclidean correlation theory

To establish a safety evaluation model, the classic grey Euclidean correlation theory was used to measure the grey correlation between all-steel-type attached lifting scaffold safety-influencing factors and the ambiguity and incompleteness of available information. Grey system theory is a quantitative and qualitative analysis approach founded by Professor Deng Julong of Huazhong University of Science and Technology of China during the 1980s. It is applicable to any sample size and to all regularities and has the advantages of computational simplicity, compatibility with small sample sizes, and reduced requirements in terms of sample law. The approach was chosen in part to ensure complete consistency between the quantitative and qualitative analysis results.

1. Deng's grey correlation [43]
  The proposed approach can be developed in part using the following formulation:
  $$\bar{\gamma }=\frac{1}{n}\sum_{k=1}^{n}\xi (k),$$ (1)
  Where *ξ*(*k*) is the correlation coefficient. Deng's grey correlation is obtained by averaging the correlation between the comparison and reference sequences at different positions. When $\sum_{k=1}^{n}\xi (k)$ remains fixed, the $\bar{\gamma }$ will remain fixed no matter how the *ξ*(*k*) changes. If it remains fixed at all positions, the effects on the factors affecting the system differ: they can be divided into major and minor effects that are discrete and volatile. If the dynamic changes in factors are not taken into account in the research process, the conclusions formed will often be inaccurate.
2. Grey Euclidean correlation [44, 45]
  The grey Euclidean correlation degree was introduced to further improve the robustness of grey correlation theory [46] in considering the impact of different correlation factors on the degree of correlation. It is calculated as follows:
  **Step 1**: Reference and comparison sequences.
    For *n* evaluation indices and *m* evaluation objects, a reference sequence
    $$x_{0}=\left\{x_{0}(k)|k=1,2,\cdots ,n\right\}$$ (2)
    is constructed, where *x*_0_(*k*) is the standard value of the *k*-th evaluation index. A comparison sequence
    $$x_{{}_{i}}^{\prime }=\left\{x_{{}_{i}}^{\prime }(k)|k=1,2,\cdots ,n\right)$$ (3)
    where $x_{{}_{i}}^{\prime }(k)$ is the score of the *k*-th index of the *i*-th evaluation object.
  **Step 2**: Standardize the sequence.
    There are many commonly used standardized methods, including mean transformation, initial value transformation, etc. In this paper, the initial value transformation method was adopted for the processing of data information using the following formulation:
    $$x_{i}(k)=\frac{x_{{}_{i}}^{\prime }(k)}{x_{{}_{i}}^{\prime }(1)}\;i=1,2,\cdots ,m;k=1,2,\cdots ,n$$ (4)
    where $x_{i}^{\prime }(1)$ is the first data point of the reference sequence, $x_{i}^{\prime }(k)$ are the data of the reference sequence, and *x*_*i*_(*k*) are the comparison sequence data following transformation.
  **Step 3**: Calculate the absolute difference sequence.
    The absolute difference between the reference and comparison sequences, *x*_0_(*k*) and *x*_*i*_(*k*), respectively, is given by
    $$\Delta_{0i}=|x_{0}(k)-x_{i}(k)|\;i=1,2,\cdots ,m;k=1,2,\cdots ,n.$$ (5)
    The absolute difference sequence matrix can then be formed as follows:
    $${\left(\begin{array}{cccc} \Delta_{11} & \Delta_{12} & \cdots & \Delta_{1n}\\ \Delta_{21} & \Delta_{22} & \cdots & \Delta_{2n}\\ \vdots & \vdots & \ddots & \vdots \\ \Delta_{m1} & \Delta_{m2} & \cdots & \Delta_{mn} \end{array}\right)}_{m\times n}.$$ (6)
    The maximum and minimum differences in the absolute difference sequence matrix are defined as Δ_max_ and Δ_min_, respectively.
  **Step 4**: Calculate the correlation coefficient [44].
    The correlation coefficient *ξ*_0*i*_ is obtained as
    $$\xi_{0i}=\frac{\Delta_{\text{min}}+\rho \Delta_{\text{max}}}{\Delta_{0i}+\rho \Delta_{\text{max}}}\;\rho \in (0,1),i=1,2,\cdots ,m,$$ (7)
    where *ρ*(0 < *ρ* < 1) is the resolution coefficient. Its value is obtained by setting *Δ*_v_ as the average of all absolute values, that is,
    $$\Delta_{\text{v}}=\frac{1}{nm}\sum_{i=1}^{m}\sum_{k=1}^{n}\left|x_{0}(k)-x_{i}(k)\right|,$$ (8)
    Which gives $\varepsilon_{\Delta }=\frac{\Delta_{v}}{\Delta_{\text{max}}}$, ε_*Δ*_ ≤ *ρ* ≤ 2*ε*_*Δ*_.
    At the same time,
    $$\Delta_{\text{max}}>3\Delta_{\text{v}}时,\varepsilon_{\Delta }<\rho \leq 1.5\varepsilon_{\Delta }$$ (9)
    $$\Delta_{\text{max}}\leq 3\Delta_{\text{v}}时,1.5\varepsilon_{\Delta }<\rho \leq 2\varepsilon_{\Delta }$$ (10)
  **Step 5**: Calculate the grey Euclidean correlation [40].
    The grey Euclidean correlation is calculated as
    $$\gamma_{0i}=1-\frac{1}{\sqrt{n}}{\left[\sum_{k=1}^{n}{\left\{\xi_{0i}(k)-1\right\}}^{2}\right]}^{1/2},$$ (11)
    where *ξ*_*oi*_(*k*) is the correlation coefficient.
  **Step 6**: Calculate the grey relative Euclidean weighted correlation.
    The grey relative Euclidean weighted correlation is based on the existing grey Euclidean correlation coefficient and used to obtain the Euclidean weighted correlation degree. It is calculated as follows:
    1. The grey weighted correlation is calculated as
      $$\gamma_{0i}=\sum_{k=1}^{n}\left[w_{i}(k)\xi_{0i}(k)\right],$$ (12)
      where *γ*_0*i*_ is the grey weighted correlation degree, *ξ*_*oi*_(*k*) is the correlation coefficient, and *w*_*i*_(*k*) is the comprehensive weight corresponding to the correlation coefficient.
    2. The grey relative Euclidean weighted correlation is then obtained by expanding the deformation around Eq (12) to form ${\bar{\gamma }}_{0i}$, that is,
      $$\bar{\gamma_{0i}}=1-{\left[{\left(\gamma_{0i}-1\right)}^{2}+\sum_{k=1}^{n}w_{i}(k)\varepsilon^{2}{}_{0i}(k)\right]}^{1/2},$$ (13)
      where *ε*_0*i*_ is the fluctuation of each correlation coefficient, *ξ*_0*i*_, with respect to the correlation degree, *γ*_0*i*_, obtained from Eq (12) and is calculated as *ε*_0*i*_(*k*) = *ξ*_0*i*_(*k*) − *γ*_0*i*_.
  **Step 7**: Relevance analysis.
    The grey relative Euclidean weighted correlation degree obtained from Eq (13) is then used to sort the evaluation items according to the grey correlation criterion [47, 48] that a greater degree of correlation will correspond to a better evaluation result.

#### 2.3.2 Determination of index weights

Primary index weight.
  The weight of the first-level indicator is an importance weight that should be determined by experts. However, the knowledge level differs by expert, which can easily lead to deviations in the results. Therefore, in this study the MABM method [49] was used to determine the weights of the five primary indicators, as this method takes the credibility of assessing experts into consideration and is more objective. The first-level indicator index weight was calculated as follows:
  **Step 1**: Determine the comprehensive credibility of each expert [49].
    The credibility of the ranking experts was assessed based on three factors: professional title, academic qualifications, and seniority. The specific scoring standards are listed in Table 7.
    Each expert was given a credibility score *t* (0≤t≤1), which was obtained from *G*_*i*_(*i* = 1,2,3), the score obtained by adding the appropriate ratings listed in Table 7 corresponding to their title, academic qualifications, and seniority as follows:
    $$t=\frac{\sum_{i=1}^{3}G_{i}}{18}.$$ (14)
    The relative ranking of each expert with respect to the other experts was then calculated as $\beta_{j}=\begin{array}{c} t_{j}/\sum_{j=1}^{m}t_{j} \end{array}$.
  **Step 2**: Expert scoring rules.
    The ranking scoring method was then used to determine the expert scoring rules. The *m* experts were sorted in terms of *n* indicators according to relative indicator importance, with *n* points given to the most important indicator and one point given to the least important and the remaining indicators scored within this range but with each score used only once. Based on this ranking, the following expert weight scoring matrix, *C*_*m*×*n*_, was obtained:
    $$C_{m\times n}=[\begin{array}{cccc} c_{11} & c_{12} & \cdots & c_{1n}\\ c_{21} & c_{22} & \cdots & c_{2n}\\ \vdots & \vdots & \ddots & \vdots \\ c_{m1} & c_{m2} & \cdots & c_{mn} \end{array}].$$
  **Step 3**: Identify the score vectors for different indicators.
    The experts’ comprehensive credit vector and the scoring matrix were multiplied together to obtain score vectors for the respective indicators, given as *w* = (*β*_1_, *β*_2_, ⋯*β*_*m*_) • *C*_*m*×*n*_ = (*w*_1_, *w*_2_, ⋯ *w*_*n*_).
  **Step 4**: Finally, the weights of different indicators were obtained using the formula $w_{i}^{\prime }=w_{i}/\sum_{i=1}^{n}w_{i}$.
Secondary index weight
  The scores of the secondary indicators were primarily obtained through scoring by the safety management personnel at the construction site. To compensate for the limited technical expertise of the scoring personnel at the construction site, an entropy weight method [43, 50] was used to determine appropriate weights based on the amount of information contained in the secondary index, a method that made full use of the original data and was relatively objective. Specifically, the structural entropy weight method was used to determine the weights of the 17 secondary indicators.
  **Step 1**: Collection of information.
    Following the procedures and requirements of the Delphi method for collecting expert opinions, an "Evaluation Index Weight Questionnaire" was developed and distributed to all experts. The anonymously completed questionnaire responses are listed in Table 8.
  **Step 2**: Expert ranking.
    The set of *k* forms filled in by *k* experts participating in the survey will correspond to an indicator set, expressed as *U* = {*u*_*1*_,*u*_*2*_,*…*,*u*_*n*_}. The sorted array matching this index set, represented as {*a*_*i*1_, *a*_*i*2_, ⋯, *a*_*in*_}, *a*_*i*1_, *a*_*i*2_, ⋯, *a*_*in*_, was assigned the positive integers {1,2, …, n} and the rank matrix A of the corresponding index was obtained through k tables. Each element of A, *a*_*ij*_, indicated the evaluation score provided by the *i*-th expert to the *j-*th index, *u*_*j*_:
    $$A=[\begin{array}{cccc} a_{11} & a_{12} & \cdots & a_{1n}\\ a_{21} & a_{22} & \cdots & a_{2n}\\ \vdots & \vdots & \ddots & \vdots \\ a_{k1} & a_{k2} & \cdots & a_{kn} \end{array}]$$
  **Step 3**: Blindness analysis.
    Definition 1: The set of positive integers *I* = 1,2…,n represents the rankings provided by an expert after evaluating the respective specific indicators using the ranking form.
      For instance, if the index *u*_*j*_ is at "first choice," then *I* = 1, etc. The number of conversion parameters, *m*, is given by *m* = *n* + 2. Then, the orders of importance *I* = *a*_*ij*_ or all experts can be sorted into
      $$\mu (I)=\begin{array}{c} \text{ln}(m-I) \end{array}/\text{ln}(m-1),$$ (15)
      where the processed conversion value *b*_*ij*_ is the membership degree of the correlation ranking number *I* and *B* is the membership matrix.
    Definition 2: Assuming that *k* experts have the same degree of authority with respect to index *u*_*j*_, the average awareness, expressed as *b*_*j*_, is given as
      $$b_{j}=(b_{ij}+b_{2j}+\cdots +b_{kj})/k.$$ (16)
    Definition 3: The implicit uncertainty in terms of expert understanding of the secondary index *u*_*j*_ is called the "cognitive blindness" and given as *σ*_*j*_(*σ*_*j*_ ≥ 0):
      $$\sigma_{j}=\sqrt{\begin{array}{cc} \frac{1}{k} & \sum_{i=1}^{k}{\left(b_{ij}-b_{j}\right)}^{2} \end{array}}.$$ (17)
    Definition 4: The overall awareness of the *k* experts with respect to each secondary index *u*_*i*_ is given as
      $$x_{j}=b_{j}(1-\sigma_{j})\;x_{j}>0.$$ (18)
      From the *x*_*j*_, the personnel evaluation vector for *u*_*j*_ can be obtained as ***X*** = (*x*_1_, *x*_2_, ⋯, *x*_*n*_).
  **Step 4**: *X* was normalized to obtain
    $$\alpha_{j}=\frac{x_{j}}{\sum_{i=1}^{n}x_{j}}.$$ (19)
    By combining the *α*_*jj*_ into the vector ***W*** = (*α*_1_, *α*_2_, ⋯, *α*_*n*_), the weight of each indicator was obtained.
3. Comprehensive weights.
  To make the weight of each secondary indicator more objective and scientific, each primary indicator weight was multiplied by the corresponding secondary indicator weight. to obtain a comprehensive secondary indicator weight ***V*** = ***w***′ • ***W***.

**Table 7:** Expert score.

Title			Education			Length of service/year		
Senior	Intermediate	Primary	PhD	Master's degree	Undergraduate	≥ 20	10–19	< 10
3	2	1	3	2	1	3	2	1

**Table 8:** Responses to expert questionnaire for determining indicator weights.

Indicator category	Evaluation expert serial number	First choice	Second choice	Third choice	Fourth choice
Indicator 1	1	√			
	2	√			
	3		√		
Indicator 2	1	√			
	2		√		
	3			√	
Indicator 3	1			√	
	2		√		
	3		√		
Indicator 4	1			√	
	2				√
	3				√

This method produces a more accurate overall ranking based on the importance rankings of each expert. The indicators designated by each expert as most, second-most, third-most important, etc., are marked with a √ in the columns corresponding, respectively, to their first, second, third, and fourth choices. It is considered equally important to allow for multiple indicators, at which point the √ marks are placed in sequence [51].

#### 2.3.3 Classification of safety evaluation level

1. Quantitative scoring standards for safety evaluation indicators.
  The cultural and technical expertise of the safety management personnel at the construction project sites were relatively low. In an actual scaffold safety evaluation process, the index quantification should be "grounded gas," that is, as simple as possible and clear and easy to implement. Given the variety of steel models, wall thicknesses, and design strengths used in different all-steel-type attached lifting scaffolds, as well as the variety of construction schemes and deformation and stability parameters, the relevant limits to the scoring standard should be obtained from the specific limits under individual construction plans and calculation books, which will not explicitly agree.
  In determining the safety evaluation index level for all-steel-type attached lifting scaffolds, the maximum and minimum standards corresponding to the "excellent" and "poor" levels, respectively, should be worked out beforehand and the interval between these should be divided into grades of good, medium, and average. In this study, the data pertaining to these five grades were derived primarily from the relevant parameters contained in the "Construction Scheme of All-steel Attachment Scaffolding Construction" document compiled from the evaluation project sites. The determination of the safety evaluation standards for the various indicators was based on the "Uniform Standards for Safety Technology for Construction Scaffolding," GB 51210 [4], and the "Steel Structure Design Standards," GB 50017 [52]. By combining these data with the results obtained by the research team from the various scaffold construction sites, a safety evaluation scoring standard for each indicator was derived. The scoring criteria are listed in Table 9.
2. Classification of safety evaluation levels
  A method for classifying the safety evaluation level of an all-steel-type attached lifting scaffold was developed by applying the grey relative Euclidean weighted correlation degree.
  Drawing on previous research results on the classification of security levels [13], the following high-to-low classification scheme was developed: Level I (high security, negligible); Level II (high security and acceptable); Level III (average safety, needs to be improved); Level IV (poor security and unacceptable)l Level V (very poor security and unacceptable). Table 10 provides a quantitative description of each safety level.

**Table 9:** Safety evaluation scoring criteria.

Indices	Evaluation level (total evaluation score C)				
	Excellent(90 ≤ C)	Good(80 ≤ C < 90)	Medium(70 ≤ C < 80)	General(60 ≤ C < 70)	Poor(C< 60)
*A*_11_	No error	There are few deficiencies	There are a few shortcomings	More dissatisfied	Mostly dissatisfied
*A*_12_	No defects	Very few defects	There are a few defects	More defects	Many defects
*A*_21_, *A*_22_, *A*_23_	Full satisfaction	Slightly above the limit	Near the limit	Slightly below the limit	Fully exceeds the limit
*A*_31_, *A*_32_, *A*_33_, *A*_34_, *A*_35_	Completely suitable	Better than the lower limit of the scheme	Approaching the lower limit of the scheme	Slightly lower than required	Severe non-compliance
*A*_41_, *A*_42_, *A*_43_, *A*_44_, *A*_51_, *A*_52_, *A*_53_	Full satisfaction	Basically satisfied	Generally satisfied	Not satisfied	Completely dissatisfied

(focus on content): *A*_11_ Corrosive wear, welding knobs, cracks, etc., *A*_12_ steel pipe usage times, *A*_21_ material stiffness crack, etc., *A*_22_ interference is too large, *A*_23_ maximum bearing capacity, *A*_31_ location and quantity, *A*_32_ distance error, *A*_33_ quantity and location, *A*_34_ location and specifications, *A*_35_ location, quantity and performance.

**Table 10:** Classification criteria for safety evaluation.

Rank	Level description	Control Strategy
Level I [0.90–1.00]	Extremely safe	Follow the current security guarantee measures and implement them steadily.
Level II [0.80–0.90)	High security	Fine local adjustment of factors affecting safety is sufficient.
Level III [0.70–0.80)	General security	Develop control measures to ensure safety and quickly resolve related issues within an effective time without delaying the progress of the operation.
Level IV [0.60–0.70)	Poor security	Work can only be continued after upgrading to level III or higher. If measures cannot be taken to ensure the safety of operations, operations must be stopped immediately.
Level V [0.00–0.60)	Extremely poor security	The safety level is extremely poor and the operation must be stopped immediately; work can be resumed after the cause has been rectified.

## 3. Results

### 3.1 Project summary

**Fig 3:**
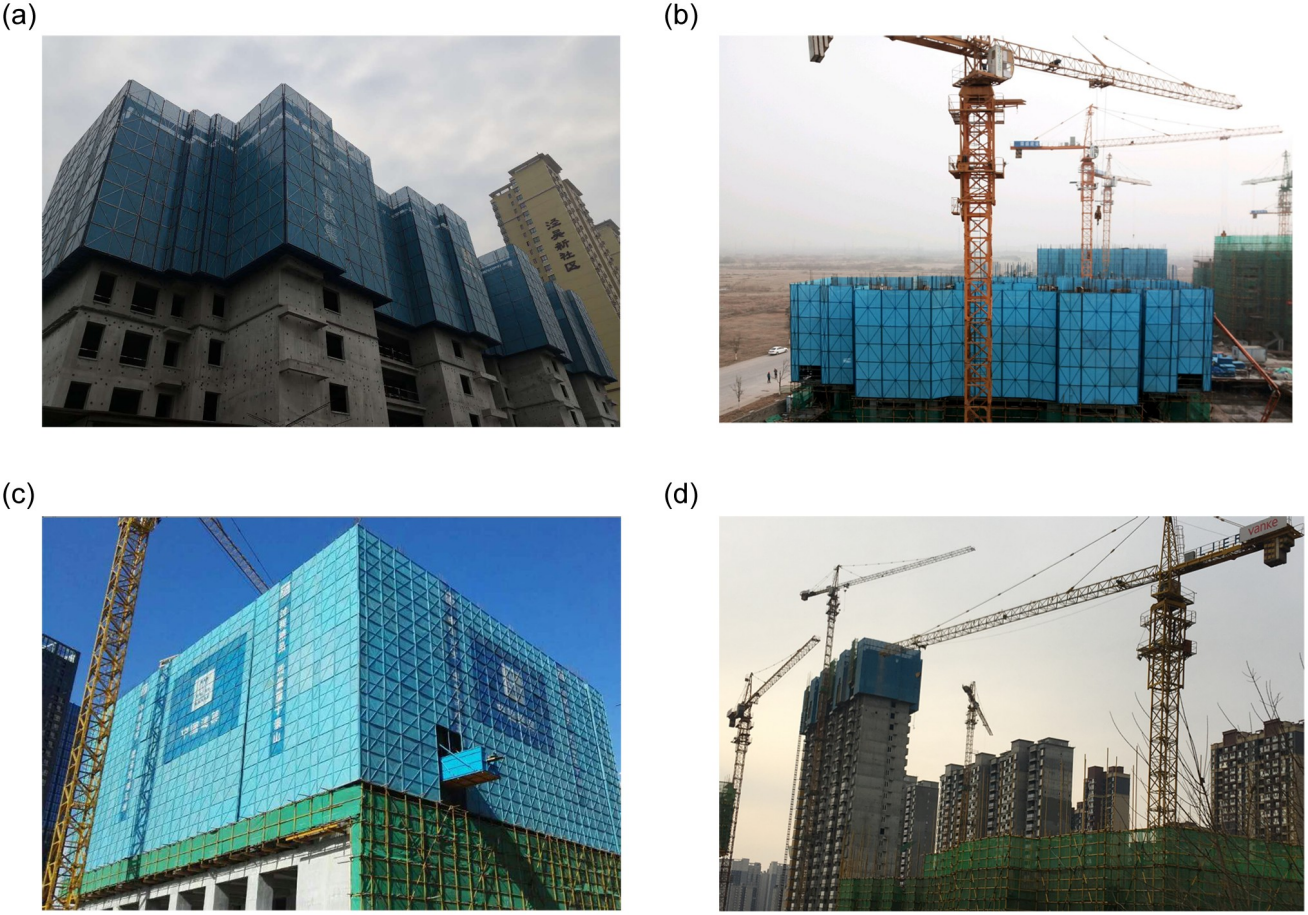
Case study scenes. (a) JWBHD project; (b) XFZY project; (c) DFCQ project; (d) CSZG6# project.

Of the 56 projects investigated in Xi'an, four of the most representative all-steel-type attached lifting scaffolding projects were selected as the research sample for this case. These are referred to here as the JWBHD, XFZY, DFCQ, and CSZG6# projects, respectively. All four used all-steel-type attached lifting scaffolds built by different companies. The case selection was representative, as shown in Fig 3.

The four projects are briefly summarized as follows:

- JWBHD project: total construction area of approximately 188,918.28 m^2^; shear wall structure; total building height 99 m; YDC-01 all-steel-type attached lifting scaffold used.
- XFZY project: total construction area approximately 239,000 m^2^; reinforced concrete frame structure; total building height 98 m; CDF-12 all-steel-type attached lifting scaffold used.
- DFCQ project: total construction area approximately 307,574.59 m^2,^; shear wall structure; total building height 99.2 m; GFD-12 all-steel-type attached lifting scaffold used.
- CSZG6 # project: total construction area 12,176.70 m^2^; shear wall structure; total building height 99.2 m; DZS-16 full-steel type attached lifting scaffolding.

### 3.2 Safety evaluation process

1. The indicator weights were determined using the following procedures.
  **Step 1**: Expert credibility.
    As described previously, the credibility of each expert was primarily measured in terms their title, educational level, and number of years of work experience. The credibility factors of the five invited experts are summarized as follows:
    - Expert 1: senior title; master's degree 25 years of work experience;
    - Expert 2: intermediate title; master's degree, 15 years of experience;
    - Expert 3: senior title, doctoral degree; 18 years of experience;
    - Expert 4: intermediate title; bachelor’s degree; 25 years of experience;
    - Expert 5: senior title; master's degree; 21 years of experience.
    Thus, for instance, the credibility score of Expert 1 was $t_{1}=\frac{3+2+3}{18}=\frac{8}{18}$. The credibility scores of other experts were obtained in the same manner.
    Within the field of five experts, Expert 1's overall credibility was
    $$\beta_{1}=\frac{\frac{8}{18}}{\frac{8}{18}+\frac{6}{18}+\frac{8}{18}+\frac{6}{18}+\frac{8}{18}}=\frac{8}{36}≅0.2222.$$
    The comprehensive credibility vector of all five experts was
    β=(0.2222,0.1667,0.2222,0.1667,0.2222).
  **Step 2**: First-level indicator weight calculation.
    The MABM method was used to determine the weights of the first-level indicators. Following the determination of the comprehensive credibility of the experts in Step 1 above, the importance scoring rules were determined and then the relative importance of each indicator was scored to determine the indicator’s weight.
    The following process was used to calculate the importance weight:
    First, the effective score vector of each evaluation object was calculated using the formula ***w*** = (*α*_1_, *α*_2_, ⋯, *α*_*m*_)***C***_*m*×*n*_:
    $$w={\left(\begin{array}{c} 0.2222\\ 0.1667\\ 0.2222\\ 0.1667\\ 0.2222 \end{array}\right)}^{\text{T}}(\begin{array}{ccccc} 3 & 2 & 5 & 4 & 1\\ 1 & 2 & 5 & 4 & 3\\ 2 & 1 & 4 & 5 & 3\\ 2 & 3 & 4 & 5 & 1\\ 1 & 3 & 5 & 4 & 2 \end{array})=(1.8333,2.1667,4.6111,4.3889,2.0000).$$
    The weight of the first-level indicator was then calculated as
    $$w_{1}=\frac{1.8333}{1.8333+2.1667+4.6111+4.3889+2.0000}=0.1222.$$
    $$w_{2}=\frac{2.1667}{1.8333+2.1667+4.6111+4.3889+2.0000}=0.1444.$$
    $$w_{3}=\frac{4.6111}{1.8333+2.1667+4.6111+4.3889+2.0000}=0.3074.$$
    $$w_{4}=\frac{4.3889}{1.8333+2.1667+4.6111+4.3889+2.0000}=0.2926.$$
    $$w_{5}=\frac{2.0000}{1.8333+2.1667+4.6111+4.3889+2.0000}=0.1333.$$
    The vector of importance weights was therefore ***w*** = (0.1222, 0.1444, 0.3074, 02926, 0.1333). The results of the importance weighting process are listed in Table 11.
  **Step 3**: Calculation of secondary indicator weights.
    The structural entropy weight method was used to determine the weights of the secondary indicators for safety evaluation. To manage the length of the discussion, here we explain only the primary calculation process. From a survey sample of 52 projects, 10 representative safety management leaders were selected. Taking material property *A*_1_ (for which *m* = 4) as an example, the matrix *A*_1_ was obtained by collating the expert questionnaire responses and then the membership matrix *B*_1_ was obtained:
    $$A_{1}=(\begin{array}{cc} 1 & 2\\ 1 & 2\\ 2 & 2\\ 2 & 1\\ 1 & 1\\ 1 & 2\\ 2 & 1\\ 2 & 2\\ 1 & 1\\ 1 & 2 \end{array}),\;B_{1}=(\begin{array}{cc} 1 & 0.6309\\ 1 & 0.6309\\ 0.6309 & 0.6309\\ 0.6309 & 1\\ 1 & 1\\ 1 & 0.6309\\ 0.6309 & 1\\ 0.6309 & 0.6309\\ 1 & 1\\ 1 & 0.6309 \end{array}).$$
    From matrix *B*_1_, *b*_1_ = 0.8524 and *b*_2_ = 0.7786.
    Based on this, the cognitive blindness of the experts on the evaluation index was *σ*_1_ = (0.1808, 0.1808). The ten personnel in charge of safety management at the sites produced an all-steel-type attachment lifting scaffold safety index evaluation vector of *X*_1_ = (0.6983, 0.6378). By applying the normalization process, a weight vector of the two secondary index under the index *u*_1_ of *W*_1_ = (0.5226, 0.4774) was obtained. A similar approach was used to obtain the weights of the remaining secondary indicators. The comprehensive weights are listed Table 12.
2. Calculation of grey relative Euclidean weighted correlation.
  Table 13 lists the scores obtained for the four research items (sites) analyzed in this study.
  The maximum and minimum absolute differences in the difference sequence matrix corresponding to these results were *Δ*_max_ = 0.210 and *Δ*_min_ = 0.030, respectively. From Eq (8), $\Delta_{\text{v}}=\frac{1}{17\times 4}\times |9.444|=0.139$ and $\varepsilon_{\Delta }=\frac{\Delta_{\text{v}}}{\Delta_{\text{max}}}=\frac{0.139}{0.210}=0.662$ were obtained. For *Δ*_*max*_ = 0.210 < 3*Δ*_*v*_, *ρ* = 1.5 *ε*_*Δ*_ = 0.993, and the *ζ*_0*i*_ were obtained from Eq (7) (Table 14).
  The grey weighted correlation degree was then obtained from Eq (12):
  $$\gamma_{o1}=[\begin{array}{l} 0.0639\times 0.677+0.0583\times 0.590+0.0569\times 0.575+0.0466\times 0.751+0.0410\times \\ 0.658+0.0822\times 0.662+0.0772\times 0.570+0.656\times 0.0511+0.665\times 0.0492+\\ 0.0478\times 0.585+0.582\times 0.0824+0.5778\times 0.0761+0.0692\times 0.694+0.645\times \\ 0.0649+0.574\times 0.0535+0.0426\times 0.606+0.0372\times 0.737 \end{array}]=0.6307,$$
  $$\gamma_{o2}=[\begin{array}{l} 0.0639\times 0.630+0.0583\times 0.696+0.0569\times 0.673+0.0466\times 0.694+0.0410\times \\ 0.630+0.0822\times 0.737+0.0772\times 0.575+0.728\times 0.0511+0.737\times 0.0492+\\ 0.0478\times 0.696+0.728\times 0.0824+0.582\times 0.0761+0.0692\times 0.756+0.597\times \\ 0.0649+0.651\times 0.0535+0.0426\times 0.677+0.0372\times 0.590 \end{array}]=0.6701,$$
  $$\gamma_{o3}=[\begin{array}{l} 0.0639\times 0.733+0.0583\times 0.682+0.0569\times 0.749+0.0466\times 0.781+0.0410\times \\ 0.682+0.0822\times 0.776+0.0772\times 0.656+0.682\times 0.0511+0.707\times 0.0492+\\ 0.0478\times 0.671+0.694\times 0.0824+0.584\times 0.0761+0.0692\times 0.761+0.591\times \\ 0.0649+0.686\times 0.0535+0.0426\times 0.619+0.0372\times 0.726 \end{array}]=0.6924,$$
  $$\gamma_{o4}=[\begin{array}{l} 0.0639\times 0.945+0.0583\times 0.892+0.0569\times 0.771+0.0466\times 0.898+0.0410\times \\ 0.742+0.0822\times 0.841+0.0772\times 0.818+0.696\times 0.0511+0.768\times 0.0492+\\ 0.0478\times 0.747+0.749\times 0.0824+0.639\times 0.0761+0.0692\times 0.988+0.622\times \\ 0.0649+0.756\times 0.0535+0.0426\times 0.660+0.0372\times 1.000 \end{array}]=0.7945.$$
  Thus, ***γ*** = (0.6307, 0.6701, 0.6924, 0.7945). The corresponding weighted correlation degree of each item was then obtained using Eq (13) (see Table 15).

**Table 11:** First-level index importance weight scores.

Expert	Expert Comprehensive Credibility	Importance score					Effectiveness score				
		*x*_1_	*x*_2_	*x*_3_	*x*_4_	*x*_5_	*x*_1_	*x*_2_	*x*_3_	*x*_4_	*x*_5_
Expert 1	0.2222	3	2	5	4	1	0.6666	0.4444	1.1110	0.8888	0.2222
Expert 2	0.1667	1	2	5	4	3	0.1667	0.3334	0.8335	0.6668	0.5001
Expert 3	0.2222	2	1	4	5	3	0.4444	0.2222	0.8888	1.1110	0.6666
Expert 4	0.1667	2	3	4	5	1	0.3334	0.5001	0.6668	0.8335	0.1667
Expert 5	0.2222	1	3	5	4	2	0.2222	0.6666	1.1110	0.8888	0.4444
Total	1	9	11	23	22	10	1.8333	2.1667	4.6111	4.3889	2.0000

**Table 12:** Weights of the indicator system.

First-level indicators	Indicator weight	Factors	Factor weight	Comprehensive weight
*A*_1_	0.1222	*A*_11_	0.5226	0.0639
		*A*_12_	0.4774	0.0583
*A*_2_	0.1444	*A*_21_	0.3937	0.0569
		*A*_22_	0.3226	0.0466
		*A*_23_	0.2837	0.0410
*A*_3_	0.3074	*A*_31_	0.2674	0.0822
		*A*_32_	0.2510	0.0772
		*A*_33_	0.1663	0.0511
		*A*_34_	0.1600	0.0492
		*A*_35_	0.1554	0.0478
*A*_4_	0.2926	*A*_41_	0.2815	0.0824
		*A*_42_	0.2601	0.0761
		*A*_43_	0.2365	0.0692
		*A*_44_	0.2218	0.0649
*A*_5_	0.1333	*A*_51_	0.4011	0.0535
		*A*_52_	0.3197	0.0426
		*A*_53_	0.2792	0.0372

**Table 13:** Summary of ideal states and expert scores for each example project.

Project Indicator	Ideal	JWBHD	XFZY	DFCQ	CSZG6#
*A*_11_	100	85.6	83.0	88.3	95.6
*A*_12_	100	80.4	86.6	85.9	94.1
*A*_21_	100	79.4	85.4	89.0	89.9
*A*_22_	100	89.1	86.5	90.3	94.3
*A*_23_	100	84.6	83.0	85.9	88.7
*A*_31_	100	84.8	88.5	90.1	92.5
*A*_32_	100	79.0	79.4	84.5	91.7
*A*_33_	100	84.5	88.1	85.9	86.6
*A*_34_	100	85.0	88.5	87.1	89.8
*A*_35_	100	80.1	86.6	85.3	88.9
*A*_41_	100	79.9	88.1	86.5	89.0
*A*_42_	100	79.5	79.9	80.0	83.5
*A*_43_	100	86.5	89.3	89.5	96.7
*A*_44_	100	83.9	80.9	80.5	82.5
*A*_51_	100	79.3	84.2	86.1	89.3
*A*_52_	100	81.5	85.6	82.3	84.7
*A*_53_	100	88.5	80.4	88.0	97.0

**Table 14:** Summary of evaluation results.

	*A*_11_	*A*_12_	*A*_21_	*A*_22_	*A*_23_	*A*_31_	*A*_32_	*A*_33_	*A*_34_	*A*_35_	*A*_41_	*A*_42_	*A*_43_	*A*_44_	*A*_51_	*A*_52_	*A*_53_
Δ_01_	0.144	0.196	0.206	0.109	0.154	0.152	0.210	0.155	0.150	0.199	0.201	0.205	0.135	0.161	0.207	0.185	0.115
Δ_02_	0.170	0.134	0.146	0.135	0.170	0.115	0.206	0.119	0.115	0.134	0.119	0.201	0.107	0.191	0.158	0.144	0.196
Δ_03_	0.117	0.141	0.110	0.097	0.141	0.099	0.155	0.141	0.129	0.147	0.135	0.200	0.105	0.195	0.139	0.177	0.120
Δ_04_	0.044	0.059	0.101	0.057	0.113	0.075	0.083	0.134	0.102	0.111	0.110	0.165	0.033	0.175	0.107	0.153	0.030
ζ_01_	0.677	0.590	0.575	0.751	0.658	0.662	0.570	0.656	0.665	0.585	0.582	0.577	0.694	0.645	0.574	0.606	0.737
ζ_02_	0.630	0.696	0.673	0.694	0.630	0.737	0.575	0.728	0.737	0.696	0.728	0.582	0.756	0.597	0.651	0.677	0.590
ζ_03_	0.733	0.682	0.749	0.781	0.682	0.776	0.656	0.682	0.707	0.671	0.694	0.584	0.761	0.591	0.686	0.619	0.726
ζ_04_	0.945	0.892	0.771	0.898	0.742	0.841	0.818	0.696	0.768	0.747	0.749	0.639	0.988	0.622	0.756	0.660	1.000
*w*_*i*_(*k*)	0.0639	0.0583	0.0569	0.0466	0.0410	0.0822	0.0772	0.0511	0.0492	0.0478	0.0824	0.0761	0.0692	0.0649	0.0535	0.0426	0.0372

**Table 15:** Safety evaluation level results.

Project	${\bar{\gamma }}_{0i}$	Evaluation results	Site investigation
JWBHD Project	0.6267	IV	IV
XFZY Project	0.6645	IV	IV
DFCQ Project	0.6867	IV	IV
CSZG 6# Project	0.7661	III	III

## 4. Discussion

As seen from Table 15, the safety evaluation level of the all-steel-type attached lifting scaffold at the CSZG6 # project site was grade *III*, which is generally safe but requires improvement. As other three projects had lower safety levels, we focus on the CSZG6 # project as an example for detailed analysis.

The all-steel-type attachment lifting scaffold used by the project had four primary components: a frame body part, an attachment guide and unloading system, a lifting system, and an intelligent control system.

**Fig 4:**
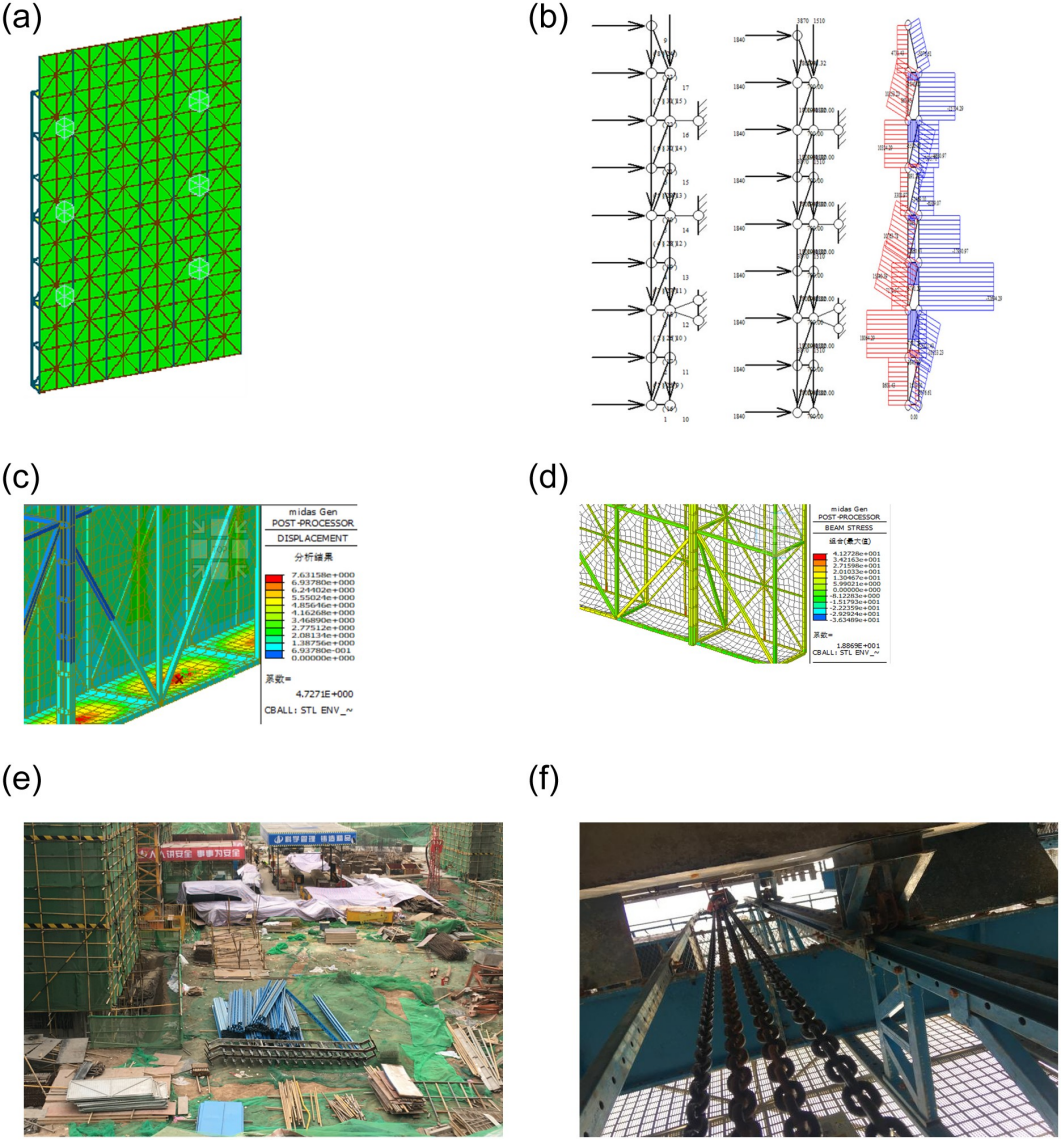
Field survey results at project CSZG6 #. (a) overall model; (b) stability analysis (wind load); (c) deformation analysis; (d) stress analysis (N / mm^2^); (e) component maintenance; (f) lifting equipment.

The height of the frame, span, and width were 13.5, 6.3, and 0.7, respectively, and the protective area of the frame was 85.05 m^2^. The guide rail was fabricated using a combination of 6.3 # channel steel back-to-back butt welding, 25-mm diameter anti-fall bar, and 10-mm steel plate. The vertical rods were all 80 × 40 × 3(unit: mm) square tubes. The non-slip steel walkway board comprised 60 × 30 × 3 (unit: mm) square tubes and 3-mm steel plate, as shown in Fig 4a.

In terms of material properties, on-site inspections carried out to determine the wall thickness and initial defects in the full-steel attached lifting scaffold square tubes and the strength of the frame revealed that all parameters met the relevant requirements under the construction scheme. In terms of structural calculation, a structural review revealed that parameters such as the maximum displacement ratio, maximum stress, and stability of the frame all met the design requirements, as shown in Fig 4b–4d.

Inspections and tests carried out to determine positions of the connecting wall and inclined bar and the platform plate, the longitudinal and step distances of the frame, and the deflection of the bar revealed that all indicators met the design requirements. A further inspection of the on-site safety management system and regular inspection systems revealed that these were all relatively complete. However, the project’s efforts in terms of safety education and training were insufficient, as primarily reflected by a lack of understanding of the mechanical performance and anti-fall functionality of all-steel-type attached lifting scaffolds and a lack of necessary safety education on the part of field safety managers and operators. In terms of equipment maintenance, main frame rails that that were subject to frequent wear lacked the necessary maintenance, while dismantled structural frames were randomly stacked on the construction site and lacked the necessary maintenance management, as shown in Fig 4e.

In the course of equipment use, materials tended to be stacked on the platform, particularly during the selection of the lifting system. The tonnage of stacked materials was just below the design requirements and the safety margin was small, as shown in Fig 4f. The above problems constituted the primary reason why the safety evaluation level of the CSZG6 # project was not Grade I.

In view of these findings, we suggest that the CSZG6 # project department should formulate corresponding safeguard measures and quickly resolve related issues within an effective time frame. If there is no improvement, the competent department should be notified to suspend operational progress to ensure the safety of the project.

The JWBHD, XFZY, and DFCQ projects all received grade IV safety evaluation grades, corresponding to poor safety levels and a need to suspend operations. The primary causes of these low ratings were similar across the three projects. In each case, it was found that the all-steel-type attached lifting scaffolding material quality control needed to be strengthened. Under the applicable standards, it is strictly forbidden to work using core members such as vertical and horizontal frames, and the material strength of the core members must be tested regularly. During the assembly and use of scaffolding, it is important to check whether the longitudinal, step, and horizontal distances of the core frame meet the relevant requirements of the construction plan and whether there are dimensional deviations caused by personnel mistakes during the construction process.

In terms of safety management, it is important that these sites strengthen the safety education and training processes, improve the comprehensive safety management system—with particular attention paid to strengthening the daily maintenance of equipment and inspection of the reliability of fall prevention devices—and formulate corresponding accident emergency plans. Projects JWBHD, XFZY, and DFCQ all need to be rectified in terms of these shortcomings. If the safety risk level can be improved to at least level III, operations can continue; if this cannot be guaranteed after taking the appropriate measures, operations must be stopped immediately.

After obtaining the safety evaluation levels of the four projects, the authors conducted field investigations and discussed the actual project situations with the relevant experts and project leaders, safety managers, and technical staff. It was agreed that the evaluation results were scientific and reasonable and consistent with the site conditions and fully reflect the safety status of the projects. This confirmation served to validate the feasibility and practicality of the proposed all-steel-type attachment lifting scaffold safety evaluation model based on grey relative Euclidean weighted correlation degree theory.

## 5. Conclusion

This paper proposed an index-based system for evaluating the safety of all-steel-type attached lifting scaffolds. The results and approach complement the existing body of research, and the use of a layered weighting system (MABM + structural entropy weight) makes the calculation results more objective and credible. The findings of this study provide a reference for future research on evaluating the safety of all-steel-type attached lifting scaffolds.

The approach described in this paper involves combining the theories of structural entropy weight and grey Euclidean weighted correlation degree to establish an all-steel-type attached lifting scaffolding safety evaluation model. Construction site-based examples were used to validate the model. The evaluation process used by the proposed model was found to be clear, operable, and highly reliable and provides a theoretical basis for relevant security management practitioners.
